# *Tricholoma matsutake* polypeptide modulates cardiac metabolism and restores autophagic flux to mitigate doxorubicin-induced senescence

**DOI:** 10.3389/fphar.2026.1842003

**Published:** 2026-08-17

**Authors:** Ting Ye, Qiu-shi Wang, Qian-wen Ge, Meng-jiao Li, Yang Yuan, Wen-she Sun, Dong-ming Xing

**Affiliations:** 1 Molecular Medicine Innovation Center, Cancer Institute of the Affiliated Hospital of Qingdao University, Qingdao University, Qingdao, China; 2 National Key Laboratory of Intelligent Tracking and Forecasting for Infectious Diseases, TEDA Institute of Biological Sciences and Biotechnology, Nankai University, Tianjin, China; 3 School of Life Sciences, Tsinghua University, Beijing, China

**Keywords:** autophagic flux, cardiac senescence, doxorubicin, metabolic reprogramming, NAD+, *Tricholoma matsutake* polypeptide

## Abstract

**Background:**

Doxorubicin (Dox)-induced cardiotoxicity (DIC) is initiated by acute stress that triggers early senescence-like phenotypes and severe metabolic maladaptation, yet effective therapeutic interventions remain elusive. *Tricholoma matsutake* polypeptide (TMP), a bioactive component from a medicinal fungus, exhibits antioxidant properties, but its potential to modulate cardiac energy metabolism remains unexplored.

**Methods:**

*In vivo*, Sprague-Dawley rats were administered Dox (20 mg/kg, i.p.) with or without TMP (200/400 mg/kg/day) for 7 days. Cardiac function was assessed via high-resolution echocardiography, and metabolic signatures were decoded using LC-MS/MS-based untargeted metabolomics. *In vitro*, H9c2 cardiomyocytes were co-treated with Dox (5 μM) and TMP (30 μg/mL). Senescence phenotypes (SA-*β*-gal), mitochondrial dynamics (JC-1/Fura-2AM), and energy metabolites (ATP/NAD+) were quantified. Mechanistic axes were interrogated via Western blotting.

**Results:**

TMP significantly attenuated Dox-induced cardiac dysfunction, interstitial fibrosis, and inflammation. Metabolomics revealed that TMP mitigated the glycolysis-dominant metabolic reprogramming, restored phospholipid homeostasis, and preserved the nucleotide pool. In cardiomyocytes, TMP antagonized premature senescence and suppressed ROS generation without compromising Dox’s antitumor efficacy in MCF-7 cells. Mechanistically, TMP alleviated the Dox-induced blockade of autophagic flux (restoring Beclin1/p-Parkin) and reactivated the AMPK/Sirt1/PGC-1α energy-sensing pathway, thereby preserving the ATP/NAD + reserve.

**Conclusion:**

TMP functions as a metabolic modulator that protects against DIC by reprogramming energy metabolism and restoring mitochondrial quality control. These findings highlight TMP as a promising adjuvant for preserving cardiovascular health in cancer survivors.

## Highlights

- TMP reverses Dox-induced cardiac senescence and dysfunction in rats.
- TMP restores the NAD + pool and activates Sirt1/AMPK/PGC-1α signaling.
- Metabolomics reveals TMP suppresses the Warburg-like glycolytic shift.
- TMP restores autophagic flux to clear damaged mitochondria.
- TMP does not compromise the antitumor efficacy of Doxorubicin.

## 1 Introduction

Anthracyclines, particularly doxorubicin (Dox), remain the cornerstone of chemotherapy for various solid tumors and hematological malignancies (Minotti et al., 2004). However, the clinical utility of Dox is severely compromised by its cumulative, dose-dependent cardiotoxicity, which can progress to irreversible dilated cardiomyopathy and heart failure, sometimes years after treatment cessation (Belham et al., 2007; Grakova et al., 2022). Historically, Dox-induced cardiotoxicity (DIC) was primarily attributed to the generation of reactive oxygen species (ROS) and the inhibition of topoisomerase IIβ, leading to cardiomyocyte apoptosis (Zhang et al., 2012). However, emerging paradigms in cardio-oncology have reframed DIC as a phenotype of therapy-induced premature senescence (Xia and Hou, 2018; Holmgren et al., 2015). Unlike acute cell death, senescent cardiomyocytes enter a state of stable cell cycle arrest while remaining metabolically active, secreting a spectrum of pro-inflammatory cytokines and matrix metalloproteinases—collectively known as the senescence-associated secretory phenotype (SASP) (Coppe et al., 2010). This microenvironment propagates damage to bystander cells, driving chronic fibrosis and functional decline (Munoz-Espin and Serrano, 2014). Thus, targeting the upstream drivers of senescence represents a novel therapeutic Frontier (Childs et al., 2015).

The heart, as the most metabolically demanding organ, relies heavily on mitochondrial plasticity to maintain contractile function (Lopaschuk et al., 2021). Recent evidence suggests that the onset of cardiac senescence is intrinsically linked to “metabolic reprogramming” and mitochondrial dysfunction (Wiley and Campisi, 2016). Under Dox stress, cardiomyocytes undergo maladaptive metabolic remodeling, characterized by disrupted lipid homeostasis and the accumulation of upstream glycolytic intermediates. Rather than effectively completing glucose oxidation, this metabolic bottleneck fails to meet the heart’s high energy demands, leading to a state of energy starvation characterized by the depletion of adenosine triphosphate (ATP) and, crucially, nicotinamide adenine dinucleotide (NAD+) (Chou et al., 2025; Abdellatif et al., 2021). As NAD+ is the obligatory substrate for Sirtuin 1 (Sirt1)—a NAD + -dependent deacetylase governing cellular longevity and mitochondrial quality control—the collapse of the NAD + pool inactivates the Sirt1/PGC-1α axis (Abdellatif et al., 2021; Gomes et al., 2013). This impairment restricts mitochondrial biogenesis and blocks the autophagic clearance of damaged organelles (mitophagy) (Barcena, 2026; Lonardo and Weiskirchen, 2026). Consequently, restoring metabolic flexibility and conserving the NAD+/ATP reserve are pivotal for mitigating the progression of energetic failure and senescence.

Current cardioprotective strategies remain inadequate (Henriksen, 2018). Dexrazoxane, the sole FDA-approved cardioprotectant for DIC, is limited by clinical concerns regarding its potential to interfere with antitumor efficacy and induce secondary malignancies (Swain et al., 1997). Consequently, there is an urgent unmet need for safe, bioactive agents that can specifically modulate cardiac energy metabolism (Kong et al., 2025). *Tricholoma matsutake* (TM), a highly prized edible fungus known as the “King of Fungi,” has been utilized in traditional medicine for millennia to enhance vitality and longevity (Zhou et al., 2023). While the immunomodulatory properties of TM polysaccharides are well-documented, the pharmacological potential of *Tricholoma matsutake* polypeptide (TMP) remains largely unexplored (Liang et al., 2025). Bioactive peptides are increasingly recognized for their ability to modulate intracellular signaling and metabolic pathways with high specificity and low toxicity (Fosgerau and Hoffmann, 2015).

In this study, we hypothesized that TMP functions as a metabolic modulator to protect against DIC. By integrating LC-MS/MS-based untargeted metabolomics with molecular mechanistic assays, we provide evidence that TMP attenuates Dox-induced cardiac senescence and structural remodeling. Specifically, we demonstrate that TMP exerts substantial metabolic modulation by suppressing maladaptive glycolysis, preserving the NAD + energy reserve, and reactivating the AMPK/Sirt1/PGC-1α signaling axis to restore autophagic flux and mitochondrial quality control.

## 2 Materials and methods

### 2.1 Chemicals and reagents

*Tricholoma matsutake* polypeptide (TMP), isolated as a standardized mixture of bioactive peptides with an overall peptide purity of >98% (excluding carbohydrates and lipids) was synthesized and supplied by Xi’an EnTaiYuan Biotech Co., Ltd. (Cat. No. ETYSW201015, Xi’an, China). Doxorubicin hydrochloride (Dox, MW: 579.98 g/mol) and Dexrazoxane (Dexra, MW: 268.27 g/mol) were purchased from Widely Chemical Technology (Hubei, China). Dulbecco’s Modified Eagle Medium (DMEM, Cat. No. PWL103), fetal bovine serum (FBS, Cat. No. PWL112), penicillin-streptomycin solution (Cat. No. PWL062), and 0.25% Trypsin-EDTA (Cat. No. PWL085) were obtained from Meilunbio (Dalian, China). Primary antibodies against PGC-1α (Cat. No. A19674), Sirt1 (Cat. No. A28514), AMPKβ1 (Cat. No. A4344), p62 (Cat. No. A19700), LC3B (Cat. No. A19665), Beclin1 (Cat. No. A7353), Bcl-2 (Cat. No. A11313), and IL-1β (Cat. No. A16288) were purchased from Abclonal (Wuhan, China). HRP-conjugated Goat Anti-Rabbit IgG (Cat. No. AS014) and HRP-conjugated β-tubulin (Cat. No. AC028) were also from Abclonal. All other analytical grade chemicals were supplied by Sinopharm Chemical Reagent Co., Ltd. unless otherwise stated.

### 2.2 Animal ethics and experimental design

Male Sprague-Dawley (SD) rats (10–12 weeks old, 300–330 g) were purchased from SPF (Beijing) Biotechnology Co., Ltd. (License No. SCXK (Jing) 2019-0010). The animals were housed in the SPF facility of Qingdao University under controlled conditions (22 °C ± 2 °C, 12 h light/dark cycle) with *ad libitum* access to food and water. All procedures complied with the NIH Guide for the Care and Use of Laboratory Animals and were approved by the Animal Ethics Committee of Qingdao University (Approval No. 20201203C577620211231069). Following a 7-day acclimatization, twenty rats were randomly divided into four groups (n = 5 per group). The Control group received a saline vehicle. The Dox group received a single intraperitoneal (i.p.) injection of Dox (20 mg/kg) on Day 4. This specific dose was selected based on established preclinical models of acute Dox-induced cardiotoxicity, as it reliably and rapidly induces severe metabolic collapse and early senescence-like phenotypes within a short timeframe. The Dox + TMP-L group was administered TMP (200 mg/kg/day) by gastric gavage for 7 consecutive days (Days 1–7), with Dox injection on Day 4. The Dox + TMP-H group was administered TMP (400 mg/kg/day) by gastric gavage for 7 consecutive days, with Dox injection on Day 4. The dose levels of TMP were determined using the body surface area (BSA) normalization method to mirror realistic translational intake (Reagan-Shaw et al., 2008). On Day 8, cardiac function was assessed, after which rats were euthanized with an overdose of pentobarbital sodium (300 mg/kg, i.p.) for tissue collection. This 7-day high-dose Dox regimen is an established acute cardiotoxicity model optimal for capturing early metabolic maladaptation and structural remodeling (Wang et al., 2009).

### 2.3 Echocardiography

Cardiac function was evaluated using a Vevo® 2100 high-resolution imaging system (VisualSonics, Toronto, Canada) equipped with an MS-250 transducer. Rats were anesthetized with 3% isoflurane for induction and maintained at 1.5%–2% isoflurane via a nose cone. Hair was removed from the chest, and ultrasound coupling gel was applied. Parasternal long-axis views were acquired in B-mode to guide M-mode recordings at the level of the papillary muscles. Left ventricular ejection fraction (LVEF), fractional shortening (LVFS), interventricular septum thickness (IVS), and left ventricular posterior wall thickness at end-systole (LVPWs) were calculated from at least three consecutive cardiac cycles by an investigator blinded to the experimental groups.

### 2.4 Histological analysis and immunohistochemistry

Heart tissues were fixed in 4% paraformaldehyde, embedded in paraffin, and sectioned at 4 μm thickness. Sections were stained with Hematoxylin and Eosin (H&E) using a staining kit (Cat. No. G1003, Servicebio, Wuhan, China) for morphological assessment and Masson’s trichrome to visualize myocardial fibrosis (collagen deposition). For immunohistochemistry, deparaffinized sections were incubated with 1% hydrogen peroxide to block endogenous peroxidase, blocked with goat serum, and then incubated with anti-IL-1β antibody (1:200, Abclonal) overnight at 4 °C. After washing, sections were incubated with biotinylated goat anti-rabbit secondary antibodies and visualized using a DAB chromogen kit. Images were captured using a light microscope (Nikon, Japan), and the area percentage of collagen fibers or IL-1β positive staining was quantified using ImageJ software (NIH, USA). Serum cardiac troponin I (cTnI) levels were measured using an ELISA kit (Cat. No. SEA478Ra, Cloud-Clone Corp., Wuhan, China).

### 2.5 Cell culture and treatments

H9c2 rat cardiomyocytes (Cat. No. CRL-1446) and MCF-7 human breast cancer cells (Cat. No. iCell-h129) were obtained from ATCC and iCell (Shanghai, China), respectively. Cells were cultured in DMEM supplemented with 10% FBS and 1% penicillin/streptomycin at 37 °C in a 5% CO2 humidified atmosphere. For viability assays, cells were seeded in 96-well plates (1 × 10^4^ cells/well). Based on preliminary CCK-8 cytotoxicity screening, 30 μg/mL was selected as the optimal TMP concentration. For mechanistic and autophagic flux experiments, H9c2 cells were divided into the following primary treatment groups: Control, Dox (5 μM), TMP (30 μg/mL), and Dox + TMP (co-treatment). To rigorously monitor and quantify functional autophagic flux, parallel cultures from the experimental groups were exposed to the vacuolar H + -ATPase inhibitor Bafilomycin A1 (BafA1, 50 nM; Cat. No. S1413, Selleck, USA) for the final 4 h of the 24 h incubation window prior to protein extraction. Cells were then harvested for subsequent analysis.

### 2.6 Assessment of senescence, ROS, and mitochondrial function

Cell viability was determined using the Cell Counting Kit-8 (CCK-8, Cat. No. MA0218, Meilunbio). Cellular senescence was detected using a Senescence β-Galactosidase Staining Kit (Cat. No. C0602, Beyotime, Shanghai, China); senescent cells (stained blue) were imaged and counted. Intracellular ROS levels were measured using the DCFH-DA fluorescent probe (Cat. No. S0033S, Beyotime). After 20 min incubation at 37 °C, fluorescence intensity was analyzed by flow cytometry (CytoFLEX, Beckman Coulter). Mitochondrial membrane potential (MMP) was assessed using the JC-1 assay kit (Cat. No. C2006, Beyotime). Intracellular calcium concentration was visualized using the Fura-2 AM probe (Cat. No. S1052, Beyotime) under a fluorescence microscope. Apoptosis was evaluated using the TUNEL BrightGreen Apoptosis Detection Kit (Cat. No. A112-01, Vazyme) and Annexin V-FITC/PI Apoptosis Detection Kit (Cat. No. MA0220, Meilunbio).

### 2.7 Untargeted metabolomics analysis

Frozen heart tissues (100 mg) were ground in liquid nitrogen and homogenized in 80% cold methanol. The supernatant was collected after centrifugation (15,000 g, 20 min, 4 °C). LC-MS/MS analysis was performed on an ExionLC™ AD system coupled with a QTRAP® 6500+ mass spectrometer (SCIEX, USA). Chromatographic separation utilized a Waters XSelect HSS T3 column (2.1 × 150 mm, 2.5 μm). The mobile phases consisted of 0.1% formic acid in water (A) and 0.1% formic acid in acetonitrile (B). The gradient elution program was set as follows: 0–2 min, 2% B; 2–15 min, 2%–100% B; 15–17 min, 100% B. Mass spectrometry was operated in both positive and negative ion modes with Multiple Reaction Monitoring (MRM). Data acquisition and processing were performed using SCIEX OS Version 1.4 software. Metabolites were identified by matching retention times and mass spectra against the KEGG and HMDB databases. Differentially expressed metabolites (DEMs) were identified based on a Variable Importance in Projection (VIP) score >1 and a Student’s t-test p-value <0.05. To control the false discovery rate during multiple testing, the p-values were adjusted using the Benjamini–Hochberg (FDR) procedure.

### 2.8 Biochemical assays

Intracellular biochemical parameters were quantified using commercial assay kits according to the manufacturer’s protocols. Cardiac ATP content was measured using an ATP Assay Kit (Cat. No. S0026B, Beyotime). NAD+/NADH levels were determined using a NAD+/NADH Assay Kit with WST-8 (Cat. No. S0175, Beyotime). Lipid peroxidation was assessed by Malondialdehyde (MDA) Assay Kit (Cat. No. S0131S, Beyotime). Superoxide Dismutase (SOD) activity was measured using a Total Superoxide Dismutase Assay Kit with WST-8 (Cat. No. S0101, Beyotime). Lactate Dehydrogenase (LDH) release was quantified using an LDH Cytotoxicity Assay Kit (Cat. No. BC0685, Solarbio, Beijing, China). Protein concentrations were determined using the BCA Protein Assay Kit (Cat. No. MA0082, Meilunbio).

### 2.9 Western blot analysis

Total protein was extracted using RIPA lysis buffer (Solarbio) containing protease and phosphatase inhibitors. Equal amounts of protein were separated by SDS-PAGE and transferred onto PVDF membranes (Millipore). After blocking with 5% non-fat milk, membranes were incubated with specific primary antibodies (1:1000 dilution) overnight at 4 °C. Bands were visualized using an ECL chemiluminescence kit (Cat. No. MA0186, Meilunbio) and captured using a Vilber FX6-XT imaging system. Band intensity was quantified using ImageJ software, with β-tubulin serving as the loading control.

### 2.10 RNA extraction and quantitative real-time PCR (RT-qPCR)

Total RNA was extracted from harvested H9c2 cells using TRIzol reagent (Invitrogen, USA) according to the manufacturer’s instructions. Reverse transcription was executed using a PrimeScript™ RT Reagent Kit with gDNA Eraser (Takara, Japan). Quantitative real-time PCR was performed on a LightCycler® 480 II system (Roche, Germany) utilizing SYBR® Green Master Mix (Vazyme, China). The relative mRNA expression levels of cellular senescence markers (*p16* and *p21*) were calculated using the 2^−ΔΔCt^ method, with *GAPDH* serving as the internal reference. The specific reference primer sequences used were as follows: *p16*: Forward 5′-GGG​CAC​TGC​TGG​AAG​CCG-3′, Reverse 5′-GGG​TGG​CGG​GGT​CAC​GAA-3'; *p21*: Forward 5′-TGT​TCC​GCA​CAG​GAG​CAA​AG-3′, Reverse 5′-CTG​ACT​CCT​TGT​TCC​GCT​GC-3'; *GAPDH*: Forward 5′-GAC​ATG​CCG​CCT​GGA​GAA​AC-3′, Reverse 5′-AGC​CCA​GGA​TGC​CCT​TTA​GT-3'.

### 2.11 Statistical analysis

Data are expressed as mean ± Standard Deviation (SD). Statistical comparisons were performed using SPSS 25.0 software (IBM, USA). Differences among multiple groups were analyzed by one-way Analysis of Variance (ANOVA) followed by Tukey’s post-hoc test for pairwise comparisons. A *P*-value <0.05 was considered statistically significant. The sample size of n = 5 per group was determined based on the 3Rs (Replacement, Reduction, Refinement) principles to minimize animal use. Because the 7-day acute Dox model yields highly consistent and robust structural and metabolic phenotypes with minimal biological variance, a post-hoc power analysis confirmed that n = 5 provided greater than 80% statistical power (α = 0.05) to detect significant differences in our primary echocardiographic and metabolomic endpoints (Wang et al., 2009; Niu et al., 2016).

## 3 Results

### 3.1 TMP preserves cardiac structural integrity and function *in vivo*

**FIGURE 1:**
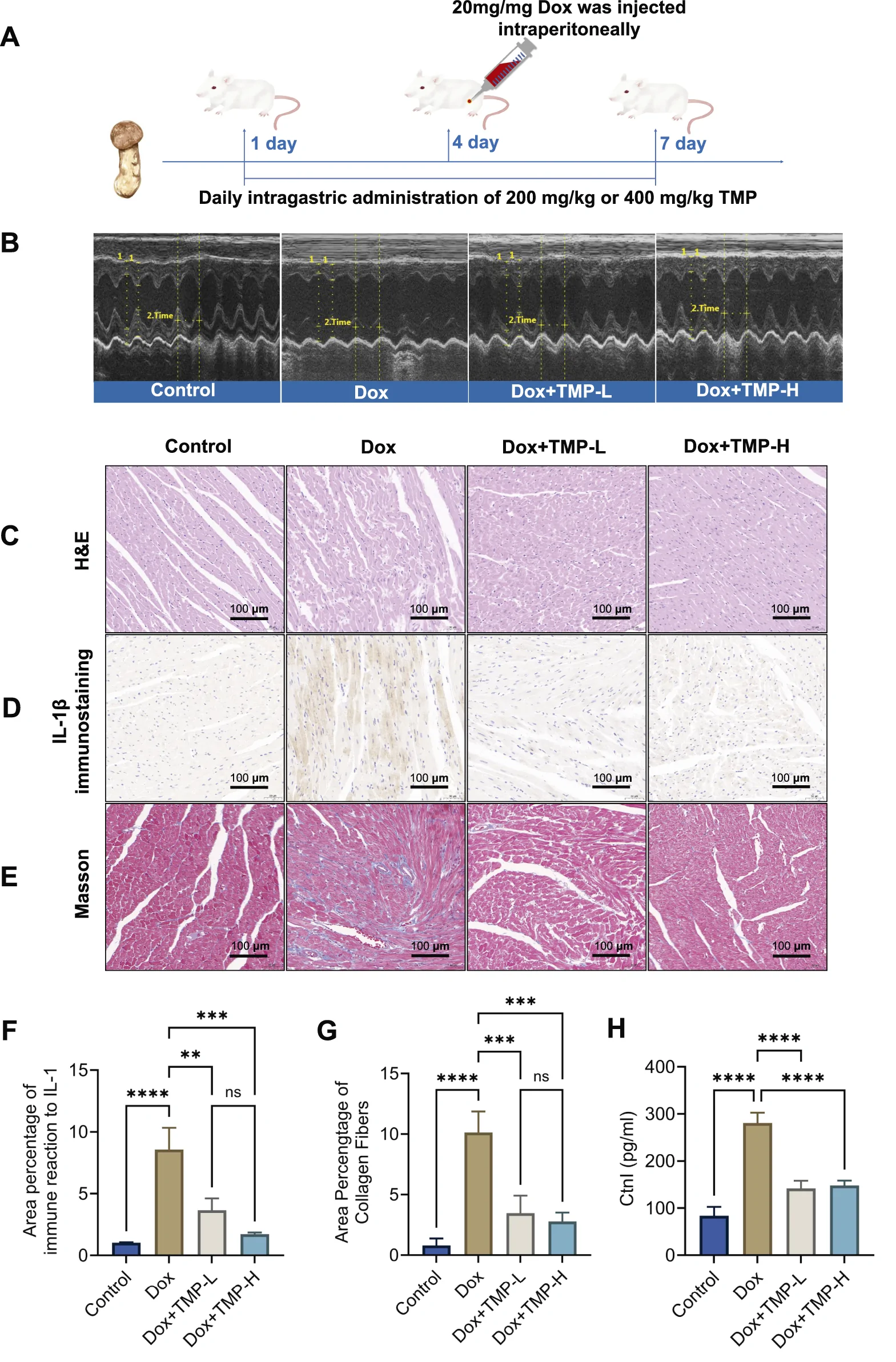
TMP mitigates Doxorubicin-induced cardiac structural damage and inflammatory response *in vivo*. **(A)** Schematic diagram of the experimental protocol. SD rats received daily intragastric administration of TMP (200 mg/kg or 400 mg/kg) for 7 days, with a single injection of Dox (20 mg/kg, i.p.) on day 4. **(B)** Representative M-mode echocardiographic images of the left ventricle, showing improved wall motion and reduced chamber dilation in TMP-treated groups. **(C)** Representative images of Hematoxylin and Eosin (H&E) staining. Scale bar: 100 μm. Dox treatment caused myofibrillar disarray and vacuolization, which were attenuated by TMP. **(D)** Representative immunohistochemical staining for IL-1β (brown) in myocardial tissue. Scale bar: 100 μm. **(E)** Representative Masson’s trichrome staining assessing fibrosis. Blue staining indicates collagen deposition. Scale bar: 100 μm. **(F–H)** Quantitative analysis of the area percentage of IL-1β positive staining **(F)**, the area percentage of collagen fibers **(G)**, and serum cardiac troponin I (cTnI) levels **(H)**. Data are presented as mean ± SD (n = 5). *, **, ***P < 0.05, 0.01, 0.001, respectively; ns, not significant (P > 0.05).

To evaluate the therapeutic potential of TMP, we established a Dox-induced cardiomyopathy model in rats according to the experimental protocol shown in Figure 1A. Echocardiographic assessment revealed severe cardiac dysfunction in the Dox group, characterized by a significant reduction in Left Ventricular Ejection Fraction (LVEF: 60% ± 3%) and Fractional Shortening (LVFS: 28% ± 2%) compared to the Control group (Table 1; Figure 1B). TMP intervention (200 mg/kg, TMP-L; and 400 mg/kg, TMP-H) significantly ameliorated these functional deficits, improving LVEF and LVFS to near-physiological levels. Histologically, H&E staining confirmed that TMP mitigated Dox-induced myofibrillar disarray and cytoplasmic vacuolization (Figure 1C). Regarding the inflammatory response, immunohistochemical staining showed elevated IL-1β expression in the Dox group, which was notably reduced in TMP-treated myocardium (Figure 1D). Furthermore, Masson’s trichrome staining qualitatively demonstrated that TMP suppressed the extensive interstitial fibrosis and collagen deposition induced by Dox (Figure 1E). Quantitative analysis corroborated these histological observations, confirming a significant reduction in the area of IL-1β positive staining (Figure 1F) and the percentage of collagen fibers (Figure 1G) in the TMP groups. Finally, consistent with the structural preservation, TMP reduced cardiomyocyte injury, as evidenced by the decreased serum cTnI levels (Figure 1H).

**TABLE 1:** Echocardiography to assess cardiac structure and function. (*P < 0.05 vs. Control, ^#^P < 0.05 vs. Dox, +P < 0.05 vs. Dox + TMP-L).

Parameters	Control	Dox	Dox + TMP-L	Dox + TMP-H
EF (%)	90.00 ± 1.00	60.00 ± 3.00	81.00 ± 10.00^#^	77.00 ± 5.00^#^
FS (%)	55.00 ± 2.00	28.00 ± 1.60	46.00 ± 12.00^#^	41.00 ± 5.00^#^
IVS (mm)	1.16 ± 0.34	0.41 ± 0.35^*^	0.62 ± 0.16^*^	0.65 ± 0.16^*^
LVPWs (mm)	3.58 ± 0.23	2.48 ± 0.42^*^	3.56 ± 0.64^#^	2.97 ± 0.46^&*^
HR (bpm)	336 ± 34.19	273.75 ± 16.98^*^	365.25 ± 28.83^#^	358 ± 48.50^#^

*P < 0.05 vs. Control, ^#^P < 0.05 vs. Dox, &P < 0.05 vs. Dox + +TMP-L.

### 3.2 TMP antagonizes senescence and oxidative stress without compromising chemotherapy

**FIGURE 2:**
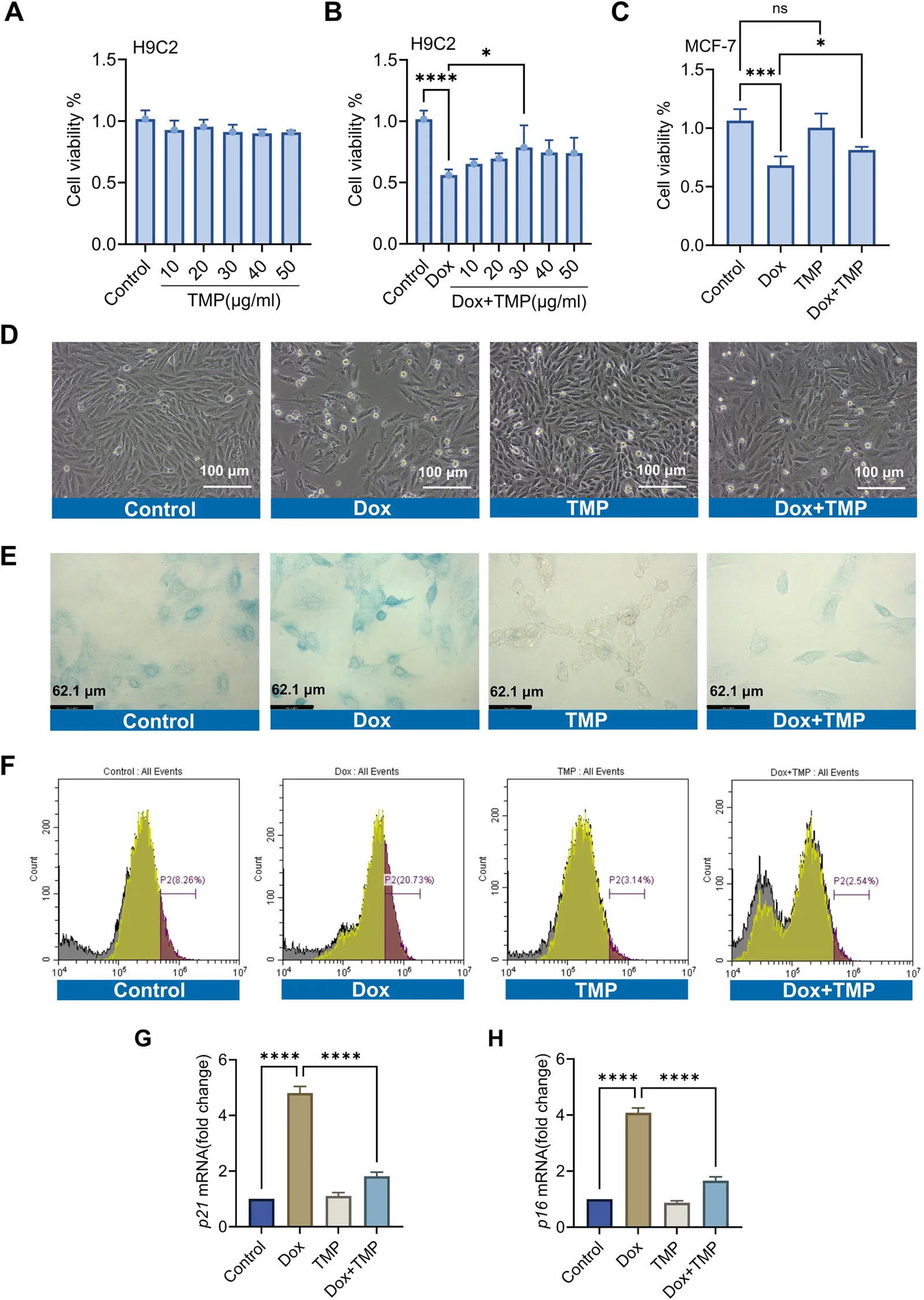
TMP protects cardiomyocytes against Dox-induced senescence and oxidative stress without compromising antitumor efficacy. **(A)** Cell viability of H9c2 cardiomyocytes treated with varying concentrations of TMP (10–50 μg/mL) for 24 h (n = 5). **(B)** Dose-response screening of TMP against Dox (5 µM)-induced injury in H9c2 cells. 30 μg/mL showed optimal protection. **(C)** Viability of MCF-7 breast cancer cells treated with Control, Dox, TMP, or Dox + TMP (n = 5). TMP did not interfere with Dox cytotoxicity. **(D)** Representative phase-contrast microscopy images of H9c2 cells showing morphological preservation by TMP. Scale bar: 100 µm. **(E)** Representative images of Senescence-Associated *β*-Galactosidase (SA-*β*-gal) staining. Blue staining indicates senescent cells. Scale bar: 62.1 µm. **(F)** Flow cytometric analysis of intracellular ROS levels using DCFH-DA staining. **(G,H)** Quantitative real-time PCR analysis of the relative mRNA expression levels of cellular senescence markers *p16* **(G)** and *p21* **(H)** in H9c2 cells. The histogram shift indicates ROS generation. Data are presented as mean ± SD. *, **, ***P < 0.05, 0.01, 0.001, respectively; ns, not significant (P > 0.05).

We next determined the optimal therapeutic window and safety profile of TMP *in vitro*. Cytotoxicity screening identified 30 μg/mL as the optimal concentration for cardio-protection (Figures 2A,B). In MCF-7 human breast cancer cells, co-treatment with TMP did not attenuate the cytotoxic efficacy of Dox (Figure 2C), suggesting a favorable safety profile for adjuvant application. Morphologically, TMP mitigated the cell shrinkage and detachment observed in Dox-treated H9c2 cardiomyocytes (Figure 2D). At the cellular level, Dox exposure induced widespread premature senescence, indicated by a robust increase in SA-*β*-gal positive staining (Figure 2E). TMP treatment significantly reduced the proportion of senescent cells. This anti-senescence effect was mechanistically linked to the suppression of oxidative stress, as TMP attenuated the Dox-induced increase in intracellular ROS levels (Figure 2F). At the transcriptional level, Dox exposure triggered a significant upregulation of the senescence-associated markers, *p16* and *p21* mRNA, compared to the Control group (Figures 2G,H). Co-treatment with TMP significantly suppressed the transcriptional activation of both *p16* and *p21*, which aligns with the morphological and SA-*β*-gal histochemical findings, indicating that TMP mitigates therapy-induced premature senescence in cardiomyocytes.

### 3.3 TMP mitigates dox-induced metabolic reprogramming

**FIGURE 3:**
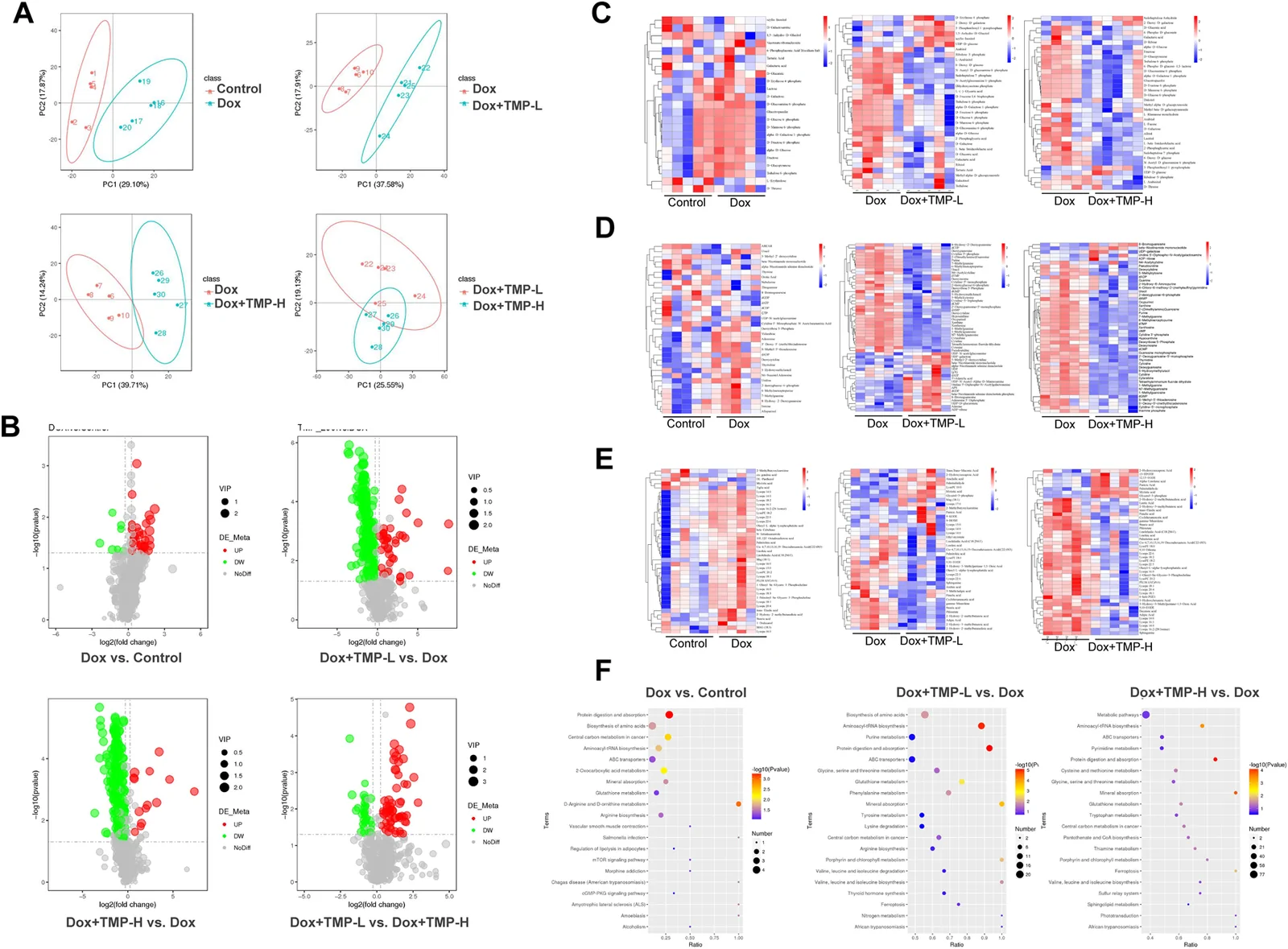
TMP reverses Dox-induced metabolic reprogramming in the rat heart. **(A)** Principal Component Analysis (PCA) score plots illustrating the separation of metabolic profiles among Control, Dox, and TMP-treated groups (n = 5). **(B)** Volcano plots visualizing significantly differentially expressed metabolites (DEMs) (Red: Upregulated; Green: Downregulated). **(C–E)** Hierarchical clustering heatmaps of differential metabolites: **(C)** Carbohydrate metabolism (showing suppression of Dox-induced glycolysis by TMP); **(D)** Nucleotide metabolism (indicating reduced free nucleotide accumulation); and **(E)** Lipid metabolism (demonstrating restoration of lipid homeostasis). **(F)** KEGG pathway enrichment analysis of DEMs, highlighting pathways related to carbon metabolism and amino acid biosynthesis.

Untargeted metabolomics was employed to characterize the metabolic signature underlying TMP-mediated protection. Principal Component Analysis (PCA) demonstrated distinct clustering between the Dox and Control groups, indicating substantial metabolic alterations (Figure 3A). Notably, TMP intervention shifted the metabolic profile of Dox-treated rats towards the Control cluster. Following the identification of differentially expressed metabolites (DEMs) (Figure 3B), hierarchical clustering was performed to visualize specific metabolic shifts. Heatmap analysis of carbohydrate metabolism revealed that Dox alters cardiomyocyte metabolism, characterized by the accumulation of upstream glycolytic intermediates (Figure 3C). We observed a significant accumulation of upstream glycolytic intermediates, such as D-Glucose 6-phosphate (log2FC = 1.53) and D-Fructose 6-phosphate (log2FC = 1.62) in the Dox group, indicating a bottleneck in glucose oxidation. TMP treatment significantly downregulated these intermediates (log2FC < −1.0), suggesting a modulation of this metabolic bottleneck. Regarding nucleotide metabolism, TMP reduced the Dox-induced accumulation of free nucleotide derivatives, indicative of improved genomic stability (Figure 3D). Furthermore, lipidomic profiling highlighted severe membrane remodeling (Figure 3E). Dox treatment led to the accumulation of lysophospholipids (e.g., Lysopc 16:1, Lysopc 18:2) and long-chain acylcarnitines (e.g., Arachidonoylcarnitine), which are associated with phospholipase activation and altered fatty acid metabolism. TMP treatment significantly reduced these lipotoxic species and normalized carnitine profiles. Finally, KEGG pathway enrichment analysis showed that the metabolic modulations of TMP were primarily enriched in carbon metabolism and amino acid biosynthesis pathways (Figure 3F).

### 3.4 TMP restores mitochondrial quality control and reactivates the energy-sensing axis

**FIGURE 4:**
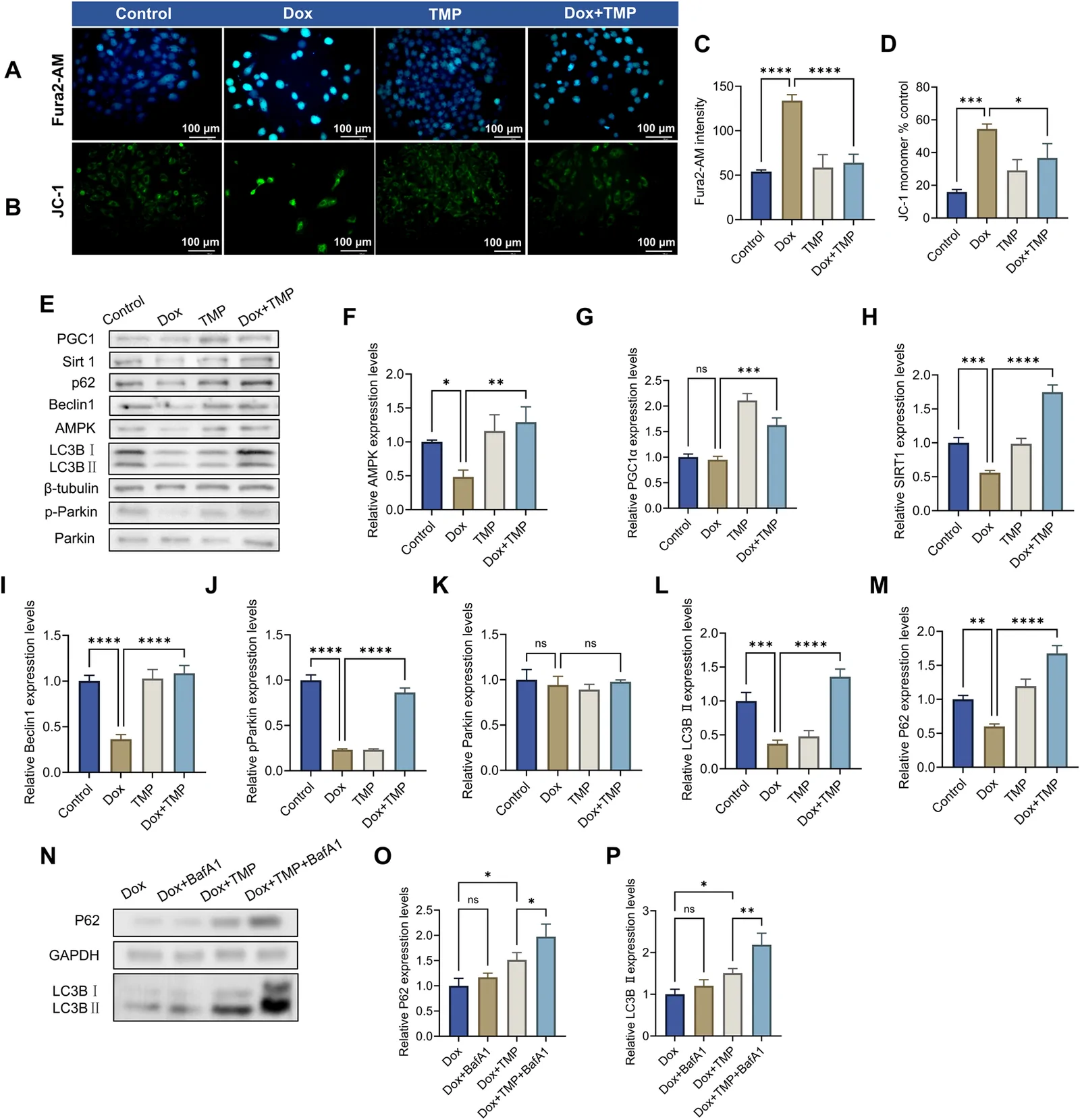
TMP restores mitochondrial quality control and activates the AMPK/Sirt1/PGC-1α signaling axis. **(A)** Representative fluorescence images of intracellular calcium levels detected by Fura-2AM staining (blue). Scale bar: 100 µm. **(B)** Representative images of mitochondrial membrane potential (ΔΨm) detected by JC-1 staining (Green: monomers/damaged). Scale bar: 100 µm. **(C,D)** Quantitative analysis of Fura-2AM fluorescence intensity **(C)** and the percentage of JC-1 monomers **(D)** (n = 3). **(E)** Representative Western blot bands showing the levels of energy metabolism-related proteins (PGC-1α, Sirt1, AMPK) and autophagy/mitophagy markers (p62, Beclin1, LC3B, Parkin, p-Parkin) in H9c2 cells treated with Control, Dox, TMP, and Dox + TMP. β-tubulin served as the loading control. **(F–M)** Quantitative densitometric analysis of relative protein expression from panel E: **(F)** AMPK, **(G)** PGC-1α, **(H)** Sirt1, **(I)** Beclin1, **(J)** p-Parkin, **(K)** Parkin, **(L)** LC3B-II, and **(M)** p62. **(N)** Representative Western blot bands for p62 and LC3B in the autophagic flux assay (cells treated with/without BafA1, 50 nM for 4 h). GAPDH served as the loading control. **(O,P)** Quantitative densitometric analysis of **(O)** p62 and **(P)** LC3B-II relative expression levels from the flux assay. Data are presented as mean ± SD (n = 3). *P < 0.05, P < 0.01, ***P < 0.001, ****P < 0.0001; ns, not significant (P > 0.05).

To elucidate the molecular mechanism, we examined the impact of TMP on cellular energy status and mitochondrial function. Biochemically, Dox treatment led to the depletion of ATP and NAD+ (a critical coenzyme for Sirt1), as well as decreased antioxidant capacity (SOD) and increased lipid peroxidation products (MDA) (Table 2). TMP significantly replenished the cellular ATP and NAD+ pools and mitigated oxidative stress. At the organelle level, fluorescence imaging revealed that Dox induced severe intracellular calcium overload and mitochondrial membrane depolarization (Figures 4A,B). TMP intervention significantly attenuated this calcium accumulation (Figure 4C) and effectively preserved the mitochondrial membrane potential (ΔΨm) (Figure 4D).

**TABLE 2:** Content detection of substances related to oxidative stress and energy metabolism.

Parameters	Control	Dox	TMP	TMP + Dox
MDA (nmol/mg protein)	0.95 ± 0.01	1.60 ± 0.19*	1.33 ± 0.01	1.31 ± 0.027^#^
SOD (U/mg protein)	39.10 ± 1.47	19.23 ± 0.86*	22.92 ± 3.82	44.88 ± 3.19^#^
ATP (nmol/mg protein)	33.65 ± 0.71	12.44 ± 0.82*	42.60 ± 1.90	33.41 ± 0.99^#^
NAD + (pmol)	1.50 ± 0.20	0.44 ± 0.11*	1.09 ± 0.11	1.71 ± 0.14^#^
LDH release (U/mL)	20.70 ± 0.14	30.86 ± 0.43*	18.78 ± 0.24	26.79 ± 1.13^#^

*P < 0.05 vs. Control, ^#^P < 0.05 vs. Dox

Western blotting revealed that TMP reactivated the energy-sensing network, as evidenced by the synchronized upregulation of AMPK, PGC-1α, and Sirt1, which were otherwise suppressed under Dox stress (Figures 4E–H). Regarding mitochondrial quality control, Dox treatment blocked the initiation of mitophagy, indicated by the downregulation of Beclin1 and p-Parkin (Figures 4I,J). Notably, while total Parkin levels remained unaltered across all experimental groups (Figure 4K), Dox induced a severe deficit in baseline LC3B-II and p62 levels (Figures 4L,M). TMP intervention significantly preserved the expression of these essential mitophagic regulators.

To determine whether the alterations in LC3B and p62 reflected changes in functional autophagic degradation rather than a static block, we performed an autophagic flux assay using the lysosomal inhibitor Bafilomycin A1 (BafA1) (Figure 4N). As shown in Figures 4O,P, the addition of BafA1 (50 nM, 4 h) to the Dox-treated group did not lead to a significant further accumulation of either p62 or LC3B-II compared to cells treated with Dox alone. This lack of additive accumulation indicates that Dox suppresses autophagic flux upstream. In contrast, in the Dox + TMP group, the introduction of BafA1 resulted in a significant further accumulation of both p62 and LC3B-II compared to the group treated with Dox + TMP without inhibitor. This additive accumulation demonstrates that TMP maintains an active and functional autophagic flux under Dox stress.

## 4 Discussion

The “energy starvation” hypothesis posits that the failing heart in Doxorubicin-induced cardiotoxicity (DIC) suffers from a severe loss of metabolic flexibility and mitochondrial quality control (Lopaschuk et al., 2021; Neubauer, 2007). While traditional paradigms have focused on oxidative stress-mediated apoptosis, recent evidence suggests that DIC is fundamentally a state of premature cardiac senescence driven by metabolic maladaptation (Wiley and Campisi, 2016). In this study, we integrated untargeted metabolomics with molecular mechanistic assays to provide evidence that *Tricholoma matsutake* polypeptide (TMP) functions as a potent metabolic reprogrammer. We demonstrate that TMP attenuates Dox-induced cardiac senescence not merely by scavenging ROS, but by exerting substantial metabolic modulation—specifically by suppressing maladaptive glycolysis, replenishing the NAD + energy reserve, and restoring autophagic flux to clear dysfunctional mitochondria.

A hallmark of the failing heart is the reversion to a fetal metabolic phenotype, characterized by a reliance on glucose over fatty acids (Kolwicz et al., 2013). Our metabolomic profiling revealed that Dox shifts cardiomyocyte metabolism towards inefficient glycolysis, evidenced by the accumulation of upstream glycolytic intermediates such as glucose-6-phosphate and fructose-6-phosphate. This shift is an inefficient compensatory mechanism that fails to meet the high energetic demands of the heart, leading to ATP depletion (Grakova et al., 2022). TMP intervention effectively suppressed these glycolytic intermediates and normalized the acylcarnitine profile, particularly reducing the accumulation of long-chain species like arachidonoyl-carnitine. Although our steady-state metabolomic profiling strongly indicates a bottleneck in glucose oxidation, future studies employing isotopic tracing and real-time metabolic flux analysis are warranted to delineate the precise kinetic dynamics modified by TMP (Gibb and Hill, 2018). Nevertheless, by relieving this metabolic bottleneck and restoring lipid utilization efficiency, TMP likely redirects the heart back towards oxidative phosphorylation, thereby preventing the chronic energy deficit that arrests the cell cycle and drives senescence.

Central to this metabolic recovery is the restoration of the NAD + pool, a critical “energy currency” that links metabolism to cellular longevity (Verdin, 2015). Our data show a significant decline of NAD+ and its precursor NMN in Dox-treated hearts. NAD+ is the obligatory substrate for Sirt1, a histone deacetylase that governs mitochondrial biogenesis and suppresses the senescence-associated secretory phenotype (SASP) (Rodgers et al., 2005; Hayakawa et al., 2015). The depletion of NAD + creates a “pseudohypoxic” state that inactivates the AMPK/Sirt1/PGC-1α axis, leading to the accumulation of defective mitochondria (Gomes et al., 2013). TMP treatment elicited a significant upregulation of NMN (over 6-fold) and restored NAD + levels. While these robust metabolomic changes clearly demonstrate a restoration of the NAD+ and NMN pools, the precise molecular targets of TMP within the NAD + salvage pathway remain to be directly identified. Nevertheless, this significant restoration creates a favorable energetic environment for reactivating Sirt1. The subsequent deacetylation and activation of PGC-1α explain the observed preservation of mitochondrial membrane potential and the structural integrity of the myocardium in our *in vivo* model (Canto and Auwerx, 2009). While our current findings robustly establish an association between TMP treatment and the reactivation of this AMPK/Sirt1/PGC-1α signaling network, definitive causal reliance remains to be established. Future investigations utilizing targeted pharmacological inhibitors or genetic knockdown approaches will be pivotal in confirming these direct mechanistic dependencies (Price et al., 2012).

Furthermore, our study resolves a key controversy regarding the role of autophagy in DIC. While some reports suggest Dox induces excessive autophagy (Kobayashi et al., 2010; Rothermel and Hill, 2007), our observation of downregulated Beclin1 and p-Parkin, alongside reduced LC3B-II turnover and the lack of additive accumulation in the BafA1 flux assay, supports the view that Dox creates a blockade in the initiation of mitophagy (Li et al., 2016; Kl et al., 2016). This failure to clear damaged, ROS-generating organelles perpetuates oxidative stress and inflammation (Zhou et al., 2011). TMP treatment significantly upregulated mitophagic initiation markers and restored autophagic flux. We propose that this restoration of mitophagic capacity acts as a crucial housekeeping mechanism, attenuating the cycle of ROS generation (Kubli and Gustafsson, 2012). This upstream quality control is complemented by the significant enhancement of the non-enzymatic antioxidant shield—specifically the increase in ascorbate and glutathione observed in our metabolomics data—which directly neutralizes leakage ROS and protects the lipidome from peroxidation (Forman et al., 2009).

Clinically, the utility of cardioprotective adjuvants is often limited by their potential interference with chemotherapy (Reichardt et al., 2018; Curigliano et al., 2012). Currently, Dexrazoxane is the only FDA-approved cardioprotectant for DIC, but its clinical application is severely limited by concerns over diminished antitumor efficacy and the risk of secondary malignancies. Unlike Dexrazoxane, which functions primarily as an iron chelator, TMP operates as a systemic metabolic reprogrammer. Importantly, our results confirmed that TMP rescues the cardiac energy crisis without compromising the cytotoxic efficacy of Dox against MCF-7 breast cancer cells. This highlights a highly favorable safety profile, making TMP a highly competitive adjuvant candidate for cardio-oncology (Lyon et al., 2022). Peptide therapeutics generally exhibit lower systemic bioavailability via oral administration due to gastrointestinal enzymatic degradation, often necessitating higher initial doses in rodent models to achieve therapeutic tissue concentrations (Fosgerau and Hoffmann, 2015). Although the oral doses of TMP (200–400 mg/kg/day) in rats appear high, applying the body surface area (BSA) normalization method translates to a Human Equivalent Dose (HED) of approximately 32–64 mg/kg/day. For a 60 kg adult, this equates to roughly 1.9–3.8 g per day, which is a highly feasible and safe translational dose for an oral dietary peptide supplement (Reagan-Shaw et al., 2008). Furthermore, as a natural bioactive component derived from a widely consumed edible fungus, TMP possesses an exceptionally wide therapeutic window. Preliminary toxicological assessments indicate its median lethal dose (LD50) exceeds 5,000 mg/kg, classifying it as practically non-toxic and reinforcing its safety profile as a long-term clinical adjuvant. TMP is currently formulated as a standardized mixture of bioactive peptides (>98% overall purity). Consequently, advancing peptidomic profiling to identify the exact functional amino acid sequences represents a primary focus for our ongoing research (Dallas et al., 2015).

To bridge the gap between preclinical findings and clinical application, the boundaries of the experimental models must be carefully considered. In this study, we utilized an acute high-dose Dox rat model and H9c2 embryonic cardiomyoblasts to successfully capture the rapid onset of metabolic crisis and premature senescence. As these models may not fully recapitulate the chronic, cumulative nature of anthracycline cardiomyopathy in adult humans, subsequent translational efforts utilizing chronic low-dose *in vivo* paradigms and human induced pluripotent stem cell-derived cardiomyocytes (hiPSC-CMs) will be essential to validate the long-term clinical efficacy of TMP (Burridge et al., 2016).

In conclusion, the present study establishes TMP not merely as a traditional antioxidant, but as a potent metabolic reprogrammer against acute Dox-induced cardiotoxicity. By restoring the NAD + pool and reactivating autophagic flux, TMP efficiently mitigates the progression of energetic failure and premature senescence. Given its high translational safety and non-interference with chemotherapy, TMP represents a highly promising, novel therapeutic strategy for early intervention in cardio-oncology.

## Data Availability

The data presented in the study are deposited in the Zenodo repository, accession number/DOI: 10.5281/zenodo.21159135.
