# Supplementary material for: Tricholoma matsutake polypeptide modulates cardiac metabolism and restores autophagic flux to mitigate doxorubicin-induced senescence
Source: Front Pharmacol. 2026 Aug 17;17:1842003. doi: 10.3389/fphar.2026.1842003 (PMC13526593; doi:10.3389/fphar.2026.1842003)
Supplement: Supplementary file 1 [file DataSheet5.docx]

PGC1 100kda

SIRT1 120kda

P62 62kda

Beclin1 60kda

AMPK 50kda

LC3B 14/16kda

Parkin52kda

pParkin 52kda
